# Condylar alterations and facial growth in children with juvenile idiopathic arthritis

**DOI:** 10.1007/s00056-020-00216-8

**Published:** 2020-02-20

**Authors:** Anna-Lena Cedströmer, Anna Andlin-Sobocki, Nadjwan Abbu, Britt Hedenberg-Magnusson, Lars Dahlström, Lillemor Berntson

**Affiliations:** 1 Department of Behavioral and Community Dentistry, Institute of Odontology, Sahlgrenska Academy, University of Gothenburg, Gothenburg, Sweden; 2 Department of Orthodontics, Folktandvården Eastman Institutet, Stockholm, Stockholms län AB Sweden; 3 Department of Surgical Sciences, Oral and Maxillofacial Surgery, Uppsala University, Uppsala, Sweden; 4 Department of Dental Medicine, Section for Orofacial Pain and Jaw Function, Karolinska Institute, Huddinge, Sweden; 5 Department of Orofacial Pain and Jaw Function, Folktandvården Eastman Institute, Stockholm, Stockholms län AB Sweden; 6 Department of Women’s and Children’s Health, Uppsala University, 75185 Uppsala, Sweden

**Keywords:** Pediatrics, Juvenile rheumatoid arthritis, Cephalometry, Temporomandibular joint, Mandible, Pädiatrie, Juvenile rheumatoide Arthritis, Kephalometrie, Temporomandibulargelenk, Unterkiefer

## Abstract

**Purpose:**

The aim of this retrospective study was to evaluate facial growth in children with juvenile idiopathic arthritis (JIA) by means of lateral head cephalometric radiographs and relate the findings to temporomandibular joint (TMJ) condylar changes on panoramic radiographs.

**Methods:**

Radiographic and medical records were evaluated in 65 children with JIA. Cephalometric and panoramic analyses were performed for the impact of condylar changes on facial growth. We compared children with condylar alterations, minor or major, with those without condylar alterations.

**Results:**

Based on panoramic radiographs, no condylar alterations were seen in 27 of the 65 children and condylar alterations were seen in 38 children (i.e., 23 had minor and 15 major condylar alterations). The cephalometric analyses of the children with condylar changes showed significant growth disturbances with a more retrognathic mandible (SNB; *p* = 0.03), retruded chin position (SNPog; *p* = 0.02), larger mandibular angulation (ML/NSL; *p* = 0.009) and maxillary angulation (NL/NSL; *p* = 0.03) compared with children without condylar alterations. Children with minor condylar alterations had a significantly more retruded chin position (SNPog) than those with no condylar changes (*p* = 0.04).

**Conclusions:**

Condylar changes in the TMJ, judged on panoramic radiography, in children with JIA, have impact on craniofacial growth. Even minor alterations seem to have an impact.

## Abstract (de)

**Ziel:**

Ziel dieser retrospektiven Studie war es, das Gesichtswachstum bei Kindern mit juveniler idiopathischer Arthritis (JIA) anhand von Fernröntgenseitenbildern des Kopfes zu beurteilen und die Ergebnisse mit den Veränderungen der Kiefergelenke (TMJ) auf Panoramaröntgenaufnahmen in Beziehung zu setzen.

**Methoden:**

Bei 65 Kindern mit JIA wurden Röntgen- und Krankenakten ausgewertet. Es wurden kephalometrische und Panoramaanalysen für die Auswirkungen von Kondylenveränderungen auf das Gesichtswachstum durchgeführt. Wir verglichen Kinder mit kleineren bzw. größeren Kondylenveränderungen mit Kindern ohne Kondylenveränderungen.

**Ergebnisse:**

Auf der Grundlage von Panoramaröntgenaufnahmen wurden bei 27 der 65 Kinder keine Kondylenveränderungen und bei 38 Kindern Kondylenveränderungen festgestellt (d. h. bei 23 Kindern gab es geringfügige, bei 15 größere Kondylenveränderungen). Die kephalometrischen Analysen der Kinder mit Kondylenveränderungen zeigten signifikante Wachstumsstörungen mit einem eher retrognathen Unterkiefer (SNB; *p* = 0,03), retrudierter Kinnposition (SNPog; *p* = 0,02), größerer Unterkiefer- (ML/NSL; *p* = 0,009) und Oberkieferneigung (NL/NSL; *p* = 0,03) im Vergleich zu Kindern ohne Kondylenveränderungen. Kinder mit geringfügigen Kondylenveränderungen hatten eine signifikant stärker retrudierte Kinnposition (SNPog) als Kinder ohne Kondylenveränderungen (*p* = 0,04).

**Schlussfolgerungen:**

Kondylenveränderungen im TMJ, die auf Panoramaröntgenaufnahmen bei Kindern mit JIA beurteilt wurden, haben Auswirkungen auf das Gesichtswachstum. Selbst geringe Veränderungen scheinen einen Einfluss zu haben.

## Introduction

Juvenile idiopathic arthritis (JIA) is a chronic autoimmune disease in which one or more joints, including the temporomandibular joint (TMJ), can be involved. It is the most common rheumatic disease of childhood, with an onset before the age of 16. The incidence in Sweden is 11–15/100,000 [1, 4], with higher susceptibility in girls than in boys, at a ratio of 2:1 [1].

In 1995, the International League Against Rheumatism (ILAR) classification, based on the number of active joints, clinical and laboratory features, as well as heredity, was proposed and it is currently used worldwide [22].

The TMJ may be the only joint involved and TMJ arthritis can be active with or without any symptoms [2]. In a review by Billau et al. [5], the reported prevalence of TMJ involvement ranged from 17 to 87%. The radiological methods used and the populations studied have varied between previous studies.

Methods for detection of inflammatory activity in the TMJ have been discussed. Panoramic radiography combined with clinical investigation has been used for a long time; it is simple, inexpensive, with relatively low radiation doses, but cannot detect ongoing inflammation. Magnetic resonance imaging (MRI) has become more common for detection of ongoing inflammation, but how often it can be used and availability varies. Radiological changes in the condylar articular surface have been associated with changes in the shape, function and development of the mandible. Using lateral head cephalometric analyses, several groups have showed a more retrognathic, shorter mandible and an increased open bite in children with JIA compared with healthy children [14, 15, 19, 24, 26, 27, 31]. The aim of the present study was to further evaluate the influence of TMJ condylar alterations on facial growth in a cohort of children with JIA, using panoramic radiography.

## Materials and methods

The present study included 65 children diagnosed with JIA by pediatric rheumatologists and referred to three dental specialist clinics in Sweden during an 8‑year period. The participating clinics were the Department of Surgical Sciences, Oral and Maxillofacial Surgery in Uppsala, the Orofacial Pain Specialist Clinic in Gothenburg, and the Department of Orofacial Pain and Jaw Function at Eastman Institute in Stockholm. Radiographic and medical records were scrutinized retrospectively [7]. Inclusion criteria for this study were patients who fulfilled the ILAR criteria for JIA [22] with no history of maxillofacial surgery and had at least one cephalometric and one panoramic radiographic registration. Radiographs had been performed due to clinical indication. The children were thus selected based on the two radiologic examinations. No MRI examinations were available for these children. The Orthopantomograph/Orthoceph® OP100 (MedWOW, Nicosia, Cyprus), was used in the Uppsala clinic, the Proline Dimax2/3 PCl Interface (Planmeca Oy, Helsinki, Finland) in the clinic in Stockholm, and the Orthopantomograph/Orthoceph® OP100 in 1999–2006 and Orthopantomograph/Orthoceph® OP200 (MedWOW, Nicosia, Cyprus) from 2006 in Gothenburg.

Time from onset of JIA to the time of cephalometric registration constituted the observation period. Time between panoramic radiograph and lateral cephalogram were recorded, since those were seldom performed at the same time point. Clinical variables, malocclusion, previous or ongoing orthodontic treatment, treatment with methotrexate or a biological agent at any time during observation period, and the number of medication periods, were recorded. Treatment with methotrexate or a biological agent during at least half of the first 6 months after onset of disease was regarded as one treatment period, as well as treatment during at least half of each coming year, counted separately.

### Cephalometric measurements

**Fig. 1:**
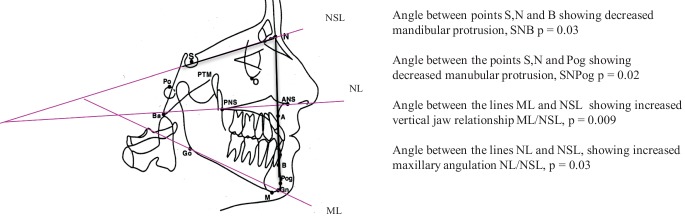
Presentation of the four measurements showing a significant difference between the 38 participants with condylar alterations compared to the 27 without condylar alterations. *SNB* angle formed by intersection of sella (S) nasion (N) and point B line; *SNPog* angle formed by intersection of sella (S), nasion (N) and point Pog line; *NSL* line through the most anterior point of the fronto nasal structure (N) and center of the sella turcica (S); *ML* mandibular line, line through Gn and the tangent point to the inferior contour, Go; *NL* nasal line, line through the apex of the anterior nasal spine (ANS) and the intersection point of the nasal floor (PNS). Modified from [9], with kind permission from Elsevier

Lateral cephalometric radiographs were recorded at a mean age of 12.0 years (standard deviation [SD] 3.1). The anteroposterior and vertical skeletal jaw relationships and mandibular incisor position were analyzed. The radiographs were taken under standardized conditions with a natural head position and the teeth with maximum intercuspation. The radiographs were analyzed with the commercially available software program FACAD (Ilexis AB, Linköping, Sweden), using standard cephalometric methods. The anatomical landmarks, lines and angles are presented in Fig. 1. The landmarks were defined in accordance with Steiner [28]. An initial calibration of the reference points was made by two of the authors (AA‑S and NA). All tracings were made by one orthodontist (NA).

### Reproducibility of the recordings

The intraexaminer reproducibility of the cephalometric measurements was determined from duplicate recordings two months apart in 16 randomly selected radiographs. The differences between the two measurements were computed. The intraclass correlation coefficients (ICC) were between 0.84 and 0.99 for the cephalometric variables, which is considered good for repeated measurements [25].

### Condylar measurements

The evaluation of the TMJs on the panoramic radiographs was performed blind to all medical and cephalometric data. Judgments were made by one dentist (A-LC) and one experienced specialist in oral radiology and if necessary reviewed until consensus was reached [8]. Condylar structural and shape alterations were analyzed. Structural changes included erosion (area with diminished cortical density), sclerosis (increased cortical density) and subchondral cysts. Changes in the shape of the condyles included flattening (loss of smooth convexity) and osteophytes (bony process on the anterior condyle). In each individual, a dichotomous judgment was made of whether or not there was an alteration in one or both condyles. To be regarded as a substantial, i.e., major alteration, both structural and shape alterations had to be seen on at least one of the TMJs on the panoramic radiograph. Shape alterations alone were regarded as minor.

### Statistical analyses

For descriptive purposes, the means and standard deviations (SD) were given for age at onset, age at cephalometric registration, cephalometric data and disease duration. To analyze differences between groups regarding condylar alterations, the Mann–Whitney U test and the χ^2^ test were used. In further analyses of risk for facial deformity based on condylar alterations, we made receiver operator characteristic (ROC) curves. The area under the curve (AUC) was calculated with 95% confidence interval (CI), and with the following interpretations: an area of 0.5 or lower was considered as no increased risk, and area ≥0.7 meant a higher risk than coincidence. For two-tailed statistical analyses, a significance level of 5% (*p* < 0.05) was used. All analyses were performed using SPSS version 23 (SPSS Inc., Chicago, IL, USA).

This multicenter study was approved by the Regional Ethical Review Board at the University of Gothenburg, Gothenburg, Sweden (Dnr 342-07).

## Results

The study cohort consisted of 50 girls (77%) and 15 boys (23%). In Table 1, the numbers of children with and without condylar alterations and data on age at onset, age at cephalometric registration, disease duration, data on malocclusion, orthodontic treatment as well as medical treatment are given. The condylar alterations were minor in 23 and major in 15 of the 38 patients (not presented in the table). We found no statistical difference between participants with or without condylar alterations regarding disease duration but children with major condylar alterations (*n* = 15) did have longer disease duration (*p* = 0.03) compared with children with no condylar alterations (*n* = 27)(data not shown). We found no statistical difference in number of participants ever receiving treatment with methotrexate or a biological agent between those with or without condylar alterations, presented in Table 1 (*p* = 0.05). The three most frequently represented ILAR categories were persistent oligoarthritis (*n* = 20; 31%), rheumatoid factor (RF)-negative polyarthritis, (*n* = 19; 29%) and extended oligoarthritis, (*n* = 13; 20%), while the remaining categories (juvenile psoriatic arthritis 9%, RF-positive polyarthritis 5%, enthesitis related arthritis 3%, undifferentiated 1.5%, systemic 1.5%) were less common. Children with major condylar alterations (*n* = 15) belonged to the persistent oligoarticular category in 53% of cases. The mean disease duration at the time of cephalometric radiography was 5.5 years (SD 4.2 years). Mean age at the time of cephalometric registration was 12 years (SD 3.0 years) and mean time from panoramic to cephalometric registration was 1.3 years (SD 2.1 years).

**Table 1:** Condylar alterations according to panoramic radiography and clinical variables in 65 children with juvenile idiopathic arthritis, classified in accordance with the International League Against Rheumatism (ILAR) criteria [21]

	*Total group*	*No condylar alterations*	*Condylar alterations*
Number (% girls)	65 (77)	27 (77.8)	38 (76.3)
Age at onset, years, mean (SD)	6.4 (4.4)	6.9 (4.2)	5.9 (4.5)
Age at cephalometry, years, mean (SD)	12.0 (3.0)	12.0 (3.0)	11.0 (3.0)
Disease duration, years, mean (SD)	5.5 (4.2)	5.0 (4.5)	6.7 (3.5)*
Malocclusion (any type), mean (SD)	0.6 (0.5)	0.6 (0.5)	0.5 (0.5)
Orthodontic treatment (previous or ongoing), mean (SD)	0.2 (0.4)	0.3 (0.4)	0.2 (0.4)
Number of medication periods, mean (SD)*	2.0 (3.0)	2.0 (3.0)	2.0 (3.0)
Treatment with methotrexate or a biological agent ever, number (%)	36 (55.4)	17 (63.0)	19 (50.0)**

*Medication period* treatment with methotrexate or a biological agent at least half of the first 6 months after onset of disease and at least half of each coming year, counted separately.

*Comparison of disease duration using Mann–Whitney U, *p* = 0.15

**Comparison of the number of participants that had been treated with methotrexate or a biological agent ever, χ^2^ test *p* = 0.05

**Fig. 2:**
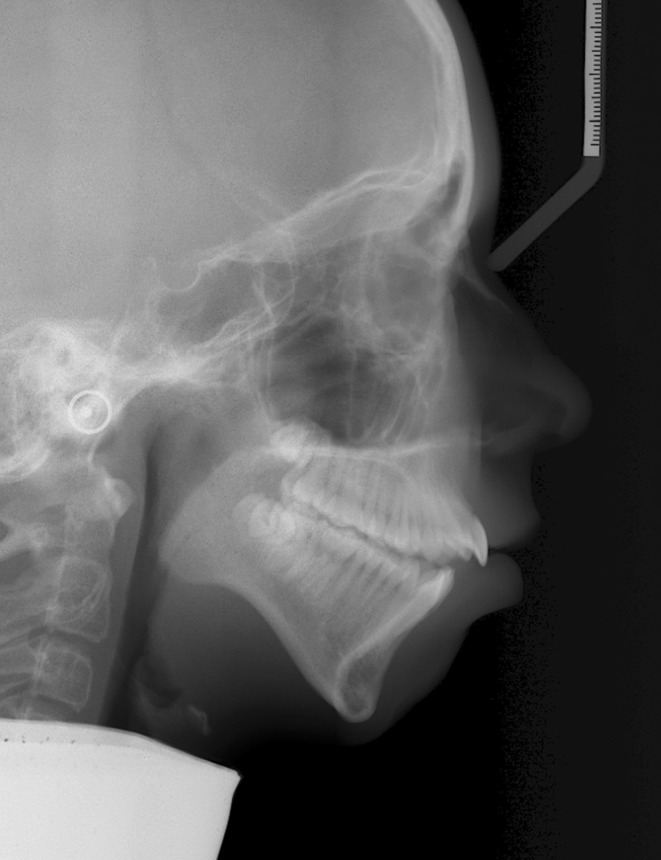
Lateral head cephalometric radiograph in a 15-year-old girl with juvenile idiopathic arthritis showing increased maxillary angulation, increased vertical jaw relationship, and decreased mandibular protrusion

**Fig. 3:**
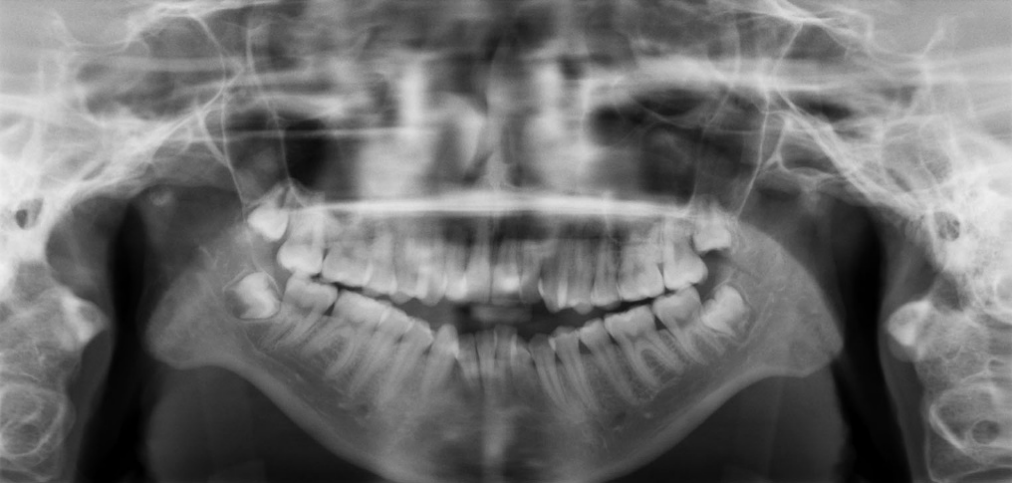
Panoramic radiograph in the same girl as in Fig. 2, showing an open bite, eroded articular bone and condylar deformity as well as impaired growth of the body and ramus mandible

**Fig. 4:**
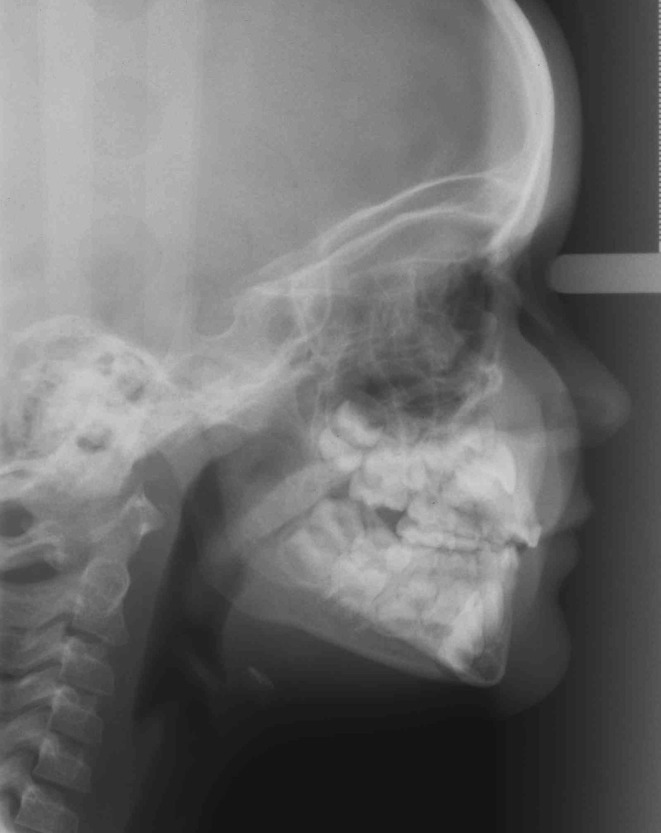
Lateral head cephalometric radiograph in a healthy teenage girl showing normal mandibular growth

**Fig. 5:**
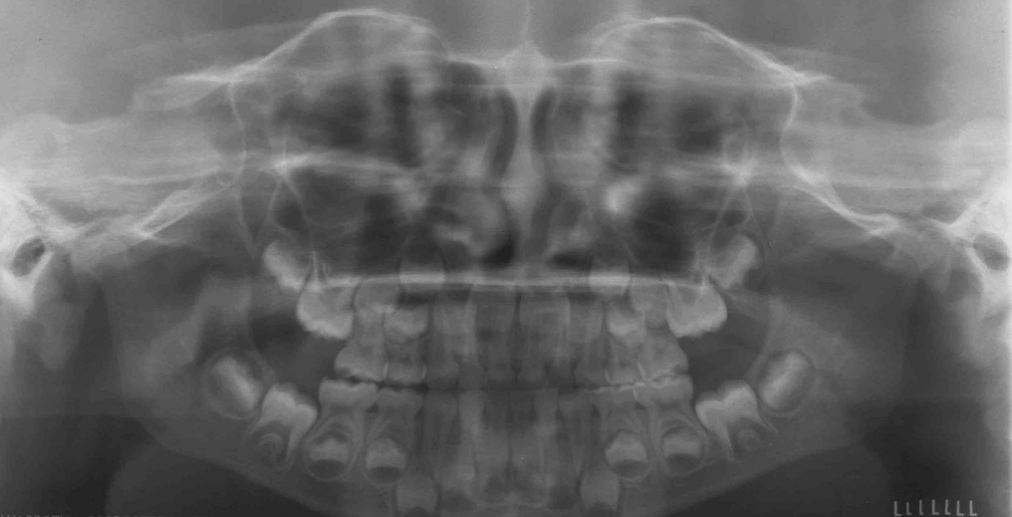
Panoramic radiograph in a healthy teenage girl showing normal condylar heads and normal bite

The results of the analyses of the cephalometric measurements in children with and without condylar alterations are given in Table 2 and Fig. 1. Children with condylar alterations (*n* = 38) showed significant growth disturbances with more retrognathic mandible (SNB; *p* = 0.03), retruded chin position (SNPog; *p* = 0.02), larger mandibular angulation (ML/NSL; *p* = 0.009) and maxillary angulation (NL/NSL; *p* = 0.03) compared with the children without condylar changes (*n* = 27). The cephalometric measurements between minor and major condylar alterations were not statistically significant (data not shown), but already with minor condylar alterations the chin position (SNPog) was significantly more retruded (*p* = 0.04) compared with in children without condylar changes. To illustrate the difference between children with JIA with condylar deformities and impaired growth of the mandible with healthy children, we have included Figs. 2, 3, 4, and 5.

**Table 2:** Cephalometric measurements in 65 children with juvenile idiopathic arthritis, divided into groups: those with no condylar alterations judged on panoramic radiographies and those with condylar alterations. Only statistically significant differences between children with condylar alterations compared with those without condylar alterations are presented

	No condylar alterations	Condylar alterations		
Variable	*n* *=27* *Mean (SD)*	*n* *=38* *Mean (SD)*	*p* value	*AUC 95% CI*
SNA	83.8 (3.1)	82.5 (3.8)	–	–
SNB	77.9 (4.4)	75.0 (4.8)	0.03	0.7 (0.5–0.8)
ANB	6.2 (2.9)	7.5 (3.6)	–	–
SNPog	78.6 (4.6)	75.5 (5.3)	0.02	0.7 (0.5–0.8)
NSBa	128.8 (5.0)	129.3 (5.9)	–	–
ML/NSL	33.1 (7.2)	37.9 (8.3)	0.009	0.7 (0.6–0.8)
NL/NSL	4.6 (3.3)	6.6 (4.3)	0.03	0.7 (0.5–0.8)
ML/NL	28.5 (6.9)	31.4 (8.1)	–	–
ILsNSL	104.9 (7.8)	102.9 (7.7)	–	–
Ili/ML	96.4 (7.7)	95.2 (7.1)	–	–
U/L FH (%)	71.5 (7.0)	72.7 (7.0)	–	–

Angle between three reference points showing maxillary protrusion (*SNA*), mandibular protrusion (*SNB*), sagittal jaw relationship (*ANB*), mandibular protrusion (*SNPog*) and cranial-base angle (*NSBa*). Angle between two reference lines showing vertical jaw relationship (*ML/NSL*), maxillary angulation (*NL/NSL*), mandibular angulation (*ML/NL*), maxillary proclination of the incisors (*ILsNSL*) and mandibular proclination of the incisors (*Ili/ML*). *U/L FH (%)* shows the relationship between two distances representing upper and lower facial height

*SD* standard deviation, *AUC* area under the curve, *95% CI* 95% confidence interval

## Discussion

The present retrospective, cross-sectional study evaluated the association between facial growth and radiographic condylar alterations of the TMJs in 65 children with JIA, mainly representing three of the ILAR categories: persistent and extended oligo arthritis, and RF-negative polyarthritis. The findings in this cohort of patients support earlier findings that condylar alterations have significant influence on facial growth, and that even minor condylar changes have an influence to some extent.

Limitations of our study were the small study cohort and the retrospective approach. Another limitation was that children were not categorized based on bilateral or unilateral condylar changes. As unilateral underdevelopment of the mandible causes chin deviations toward the affected side, a two-dimensional projection as in cephalometric radiographs may lead to underestimation. Another weakness was that time between panoramic radiography and cephalometric registration differed, and the degree of inflammatory activity during that time period was unknown.

A strength of our study is the very close retrospective work-up, and having both clinical data and radiological data. The occurrence of condylar alterations in the different categories of JIA has varied in previous studies [6, 10]. Our study did not aim to explore this question and our study cohort was small, but we found a group of children with persistent oligoarticular JIA and severe destruction in the TMJ, illustrating the spread of severe TMJ arthritis in JIA categories. The persistent oligoarticular JIA has been a category with a preferable outcome in follow-up studies [20], but it is striking that this category has not been spared in terms of condylar alterations in the TM joints. Another possible explanation could be that this group of patients most likely has had fewer periods of DMARDs (disease-modifying anti-rheumatic drugs), which may protect from TMJ arthritis. On the other hand, earlier data also raise questions about the effectiveness of systemic medical treatment on TMJ arthritis. In our study the two groups, patients with or without condylar changes did not differ regarding systemic medication and patients with condylar changes did not have a significantly longer disease duration. Intra-articular glucocorticoid injections were given very seldom in this retrospective cohort, which otherwise possibly could have explained growth impairment according to recent studies [29].

Studies of children with JIA have shown that changes in the condylar articular surface may result in changes in shape, function, and development of the mandible [14, 15, 19, 24, 31]. Erosion or resorption of the condylar head of the TMJ results in an anterior change in the position of the condyle and posterior rotation in the position of the mandible [16, 17, 27]. In the present study, characteristics of posterior rotated and retrognathic mandible analyzed on lateral cephalometric radiographs could to some extent already be seen in children with minor condylar alterations, but, most of all, condylar alterations seemed to be important risk factors for impaired facial growth. These observations are in line with a 2016 report by Hsieh et al., who also using a grading system for severity of radiological changes [12], as well as a recent report using three-dimensional assessment of dentofacial growth [30]. In the latter study, seven three-dimensional measurements were found to be the most significant for detecting impairment of facial growth; one of them was assessed in our two-dimensional study as well (ML/NSL) and found to be significant.

Previous studies have shown that the facial pattern in patients with JIA is related to the disease course and activity [11, 33]. Serial records have also shown that the facial deformity may worsen with age, and the earlier the onset of the disease, the more abnormal the subsequent mandibular development [3, 31, 32, 34, 35]. However, it is important to remember that unfavorable facial development may not be explained solely by condylar changes, as it may be prevalent among healthy children without a diagnosis of JIA [23]. Children with JIA may also have normal facial growth, despite detectable condylar lesions on panoramic radiographs [21].

Our proposed grading system for minor and major condylar alterations is very similar to a grading system previously proposed by Koos et al. [16]. In 2018, a TMJ atlas for detection and grading of juvenile idiopathic arthritis involvement by magnetic resonance imaging was presented [13]. It is important to develop and implement this further as condylar changes seem to be such an important risk factor for impaired facial growth. We are aware that panoramic radiology is an imprecise method for detecting condylar alterations in the TMJ, compared with computed tomography and MRI, but it is often the only method available.

Different methods for visualizing the TMJs in children with JIA have been used and studied through the years. As an aid to early diagnosis, MRI is considered the best method, as it can detect alterations even in early phases [18, 36]. MRI was not available in our patients. There is no one single method that fully covers ongoing inflammation and damage in the TMJ, as well as facial growth, but our study reminds us that panoramic radiology can be a useful method to screen for damage of the TMJs even though it is not very sensitive. It also tells us that even a minor condylar alteration on panoramic radiographs might indicate impaired facial growth.

## Conclusions

In this study of children with different categories of JIA, we found that even minor TMJ condylar changes, visualized by panoramic radiography, had an impact on craniofacial growth.
